# *N*-Acetylglucosamine Metabolism Promotes Survival of *Candida albicans* in the Phagosome

**DOI:** 10.1128/mSphere.00357-17

**Published:** 2017-09-06

**Authors:** Elisa M. Vesely, Robert B. Williams, James B. Konopka, Michael C. Lorenz

**Affiliations:** a Department of Microbiology and Molecular Genetics, University of Texas McGovern Medical School, Houston, Texas, USA; b Department of Molecular Genetics and Microbiology, Stony Brook University, Stony Brook, New York, USA; Carnegie Mellon University

**Keywords:** *Candida albicans*, *N*-acetylglucosamine, host-cell interactions, phagosomes

## Abstract

Phagocytosis by innate immune cells is one of the most effective barriers against the multiplication and dissemination of microbes within the mammalian host. *Candida albicans*, a pathogenic yeast, has robust mechanisms that allow survival upon macrophage phagocytosis. *C. albicans* survives in part because it can utilize the alternative carbon sources available in the phagosome, including carboxylic acids and amino acids. Furthermore, metabolism of these compounds raises the pH of the extracellular environment, which combats the acidification and maturation of the phagolysosome. In this study, we demonstrate that metabolism by *C. albicans* of an additional carbon source, *N*-acetylglucosamine (GlcNAc), facilitates neutralization of the phagosome by a novel mechanism. Catabolism of GlcNAc raised the ambient pH through release of ammonia, which is distinct from growth on carboxylic acids but similar to growth on amino acids. However, the effect of GlcNAc metabolism on pH was genetically distinct from the neutralization induced by catabolism of amino acids, as mutation of *STP2* or *ATO5* did not impair the effects of GlcNAc. In contrast, mutants lacking the dedicated GlcNAc transporter gene *NGT1* or the enzymes responsible for catabolism of GlcNAc were defective in altering the pH of the phagosome. This correlated with reduced survival following phagocytosis and decreased ability to damage macrophages. Thus, GlcNAc metabolism represents the third genetically independent mechanism that *C. albicans* utilizes to combat the rapid acidification of the phagolysosome, allowing for cells to escape and propagate infection.

**IMPORTANCE** *Candida albicans* is the most important medically relevant fungal pathogen, with disseminated candidiasis being the fourth most common hospital-associated bloodstream infection. Macrophages and neutrophils are innate immune cells that play a key role in host defense by phagocytosing and destroying *C. albicans* cells. To survive this attack by macrophages, *C. albicans* generates energy by utilizing alternative carbon sources that are available in the phagosome. Interestingly, metabolism of amino acids and carboxylic acids by *C. albicans* raises the pH of the phagosome and thereby blocks the acidification of the phagosome, which is needed to initiate antimicrobial attack. In this work, we demonstrate that metabolism of a third type of carbon source, the amino sugar GlcNAc, also induces pH neutralization and survival of *C. albicans* upon phagocytosis. This mechanism is genetically and physiologically distinct from the previously described mechanisms of pH neutralization, indicating that the robust metabolic plasticity of *C. albicans* ensures survival upon macrophage phagocytosis.

## Abstract (precis)

*Candida albicans* is the most important medically relevant fungal pathogen, with disseminated candidiasis being the fourth most common hospital-associated bloodstream infection. Macrophages and neutrophils are innate immune cells that play a key role in host defense by phagocytosing and destroying *C. albicans* cells. To survive this attack by macrophages, *C. albicans* generates energy by utilizing alternative carbon sources that are available in the phagosome. Interestingly, metabolism of amino acids and carboxylic acids by *C. albicans* raises the pH of the phagosome and thereby blocks the acidification of the phagosome, which is needed to initiate antimicrobial attack. In this work, we demonstrate that metabolism of a third type of carbon source, the amino sugar GlcNAc, also induces pH neutralization and survival of *C. albicans* upon phagocytosis. This mechanism is genetically and physiologically distinct from the previously described mechanisms of pH neutralization, indicating that the robust metabolic plasticity of *C. albicans* ensures survival upon macrophage phagocytosis.

## INTRODUCTION

*Candida albicans* is a commensal fungal species that can cause severe disseminated infections given certain medical predisposing factors, many of which are related to prolonged hospitalization, implanted medical devices, and extended antibiotic usage. *Candida* species are collectively responsible for 10 to 12% of hospital-acquired bloodstream infections, and *C. albicans* accounts for about half of these (1–3). One of the main bottlenecks that prevents dissemination of *C. albicans* is the phagocytes of the innate immune system, including macrophages and neutrophils (4). Phagocytes deploy many antimicrobial mechanisms to combat pathogens: oxidative and nitrosative stresses, a rapid influx of cations, hydrolytic enzymes optimized for the acidic environment of the phagolysosome, and a nutrient-sparse environment are characteristic of a maturing phagolysosome (4, 5). *C. albicans* has complex and dynamic responses to phagocytosis, both by neutrophils and macrophages, that enable it to resist many of these antimicrobial mechanisms. These adaptations are particularly effective in promoting fungal survival after contact with macrophages.

The resistance of *C. albicans* to the antimicrobial activities of macrophages is multifactorial and includes robust responses to reactive oxygen and nitrogen species, micronutrient scavenging, and aberrations in intracellular trafficking (reviewed in references [Bibr B6][Bibr B7][Bibr B8]. Hyphal morphogenesis is another key virulence trait of *C. albicans*, important in many stages of infection and critical to invasion of tissues and colonization (9, 10). The hyphal growth program is activated within macrophages and damages the macrophages both by physical disruption and by induction of pyroptosis, a proinflammatory cell death mechanism, which combine to allow escape of *C. albicans* (11–14).

Transcriptional and genetic studies indicate that certain host niches, including the macrophage phagolysosome, are glucose poor and that utilization of less-favored carbon sources, such as amino and carboxylic acids, are an important adaptation that promotes fitness within the host (15–18). Mutants impaired in central carbon metabolism and gluconeogenesis are attenuated for virulence (19–24). Additionally, alternative carbon compounds, such as lactate, have been shown to act as a signal that alters interactions with the mammalian host (25–27). *In vitro*, *C. albicans* utilizes these nutrients to satisfy cellular carbon requirements far more avidly than related model yeasts. Despite a lack of preferred carbon sources in these host niches, it is clear that *C. albicans* obtains adequate nutrients in order to stimulate filamentation, or hyphal morphogenesis.

Our laboratory has shown that the catabolism of amino and carboxylic acids allows *C. albicans* to neutralize acidic environments, including the phagolysosome (15, 21, 24, 28). The ability to utilize amino acids as a carbon source is dependent on the transcription factor Stp2, which controls the expression of amino acid permeases, and members of the Ato family, which are implicated in ammonia release. Genetic mutants lacking either Stp2 or Ato5 have defects in mammalian virulence (15, 24), indicative of their importance in host-pathogen interactions. Strains lacking Stp2 have a defect in hyphal morphogenesis after phagocytosis by macrophages, illustrating an important link between nutrient availability and filamentation. Importantly, amino acid catabolism and carboxylic acid catabolism are two distinct mechanisms of pH neutralization, as Stp2 is not required for this rise in ambient pH when carboxylic acids are utilized. This further highlights the adaptive ability of *C. albicans* to acidic pH and carbon starvation, as illustrated by these two distinct mechanisms to combat stresses.

Recently, it has been shown that *C. albicans* can raise the environmental pH when *N*-acetylglucosamine is the main carbon source (29). *N*-Acetylglucosamine (GlcNAc) is an amino sugar with roles in eukaryotic signaling in the mammalian host and cell wall remodeling in bacterial species, and it also comprises the basis of chitin in fungal species (30–33). In *C. albicans*, GlcNAc potently stimulates hyphal morphogenesis and also contributes to the expression of important virulence factors, such as adhesins (34–36). In this study, we aimed to determine whether GlcNAc metabolism played a role in the interactions of *C. albicans* with mammalian macrophages and whether the effects were mediated by the same pathways that were identified for the effects of amino and carboxylic acid utilization in the phagosome. The results indicate that GlcNAc utilization represents a third pathway for blocking acidification of the phagosome.

## RESULTS

### Growth with *N*-acetylglucosamine induces rapid pH neutralization.

**FIG 1:**
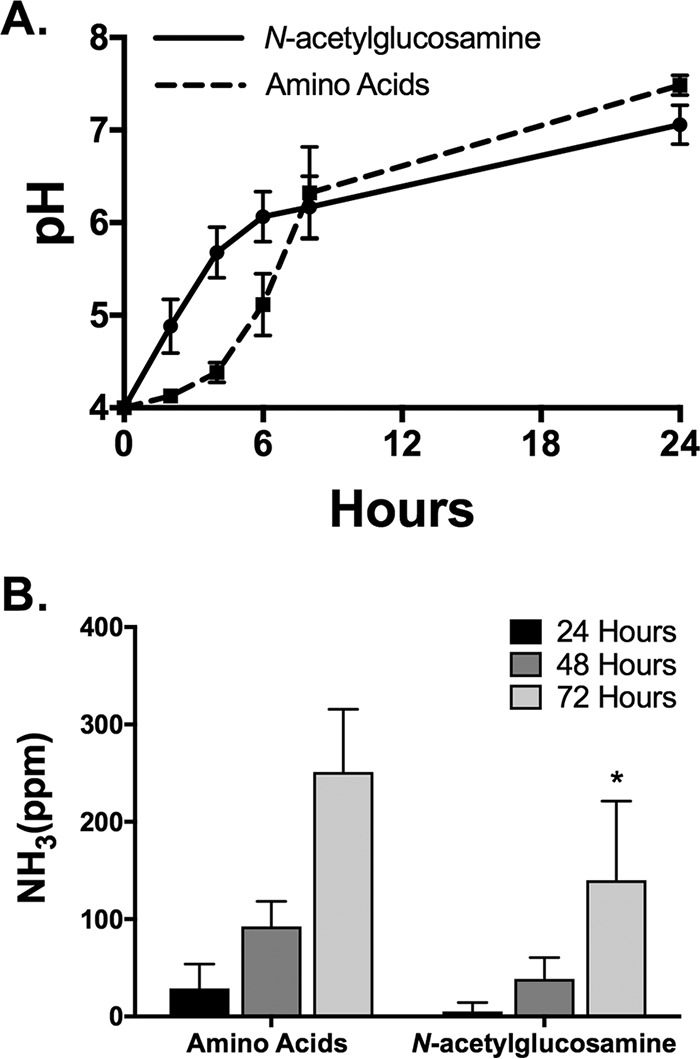
Growth with *N*-acetylglucosamine rapidly raises the environmental pH and releases detectable ammonia. (A) Wild-type *C. albicans* strain SC5314 was assessed for pH neutralization in YNBAG plus 20 mM *N*-acetylglucosamine or 1% Casamino Acids over 24 h. pH was adjusted to 4 with HCl prior to inoculation. (B) Ammonia released by *C. albicans* cells during pH neutralization on solid medium containing YNBAG plus 20 mM *N*-acetylglucosamine or 1% Casamino Acids. Ammonia was detected after 24, 48, and 72 h via Nessler’s reagent. *, *P* < 0.05. Data are expressed as mean values ± standard deviation (SD) from triplicate experiments. YNBAG medium consists of minimal YNB supplemented with 0.5% glycerol and 0.5% allantoin.

*C. albicans* can neutralize acidic environments in the presence of nonpreferred carbon sources, including amino acids and carboxylic acids, and we have demonstrated that these two phenomena are genetically and physiologically distinct (15, 28). Growth with *N*-acetylglucosamine (GlcNAc) as the sole carbon source can also promote neutralization of the ambient pH (29). We sought to further understand the physiology of this process as well as the genetic requirements to determine if this represented a third independent mechanism by which *C. albicans* neutralizes its environment. To do so, we grew *C. albicans* in minimal media with either GlcNAc or Casamino Acids (CAA) as the carbon source. These media also contained 0.5% glycerol, a carbon source that neither promotes nor inhibits pH changes (28), to minimize differences in growth rates when using mutant strains that cannot utilize GlcNAc (see below). Wild-type control cells alkalinized the medium and also excreted ammonia, as expected (28) (Fig. 1). Interestingly, cells grown in GlcNAc neutralized the acidic medium faster than those grown on CAA for the first 6 h, although the endpoint pH was roughly similar after 8 to 12 h (Fig. 1A). GlcNAc also more quickly induced large, flocculated, hyphal mats than did amino acids (not shown). Significant levels of ammonia were produced and excreted by cells grown on both media, but a smaller amount was observed in GlcNAc-grown cells, despite the higher rate of pH change (Fig. 1B). The difference in ammonia release may be related to the differences in metabolism of GlcNAc versus amino acids or buffering capacity of the media (see Discussion).

We also compared pH neutralization in GlcNAc to the phenomenon observed in the presence of carboxylic acids such as α-ketoglutarate (αKG). While the magnitude and kinetics of alkalinization of the media were similar between the two conditions, we did not detect ammonia excretion from the αKG-grown cells, nor did these cells germinate, as previously reported (15) (data not shown). Thus, the physiologies of carboxylic acid- and GlcNAc-induced pH changes are substantially different.

Utilization of GlcNAc is a trait shared with other CUG clade species, each of which is capable of neutralizing acidic environments when grown on this carbon source (see Fig. S1 in the supplemental material). In contrast, GlcNAc induces filamentation only in *C. albicans*. The GlcNAc gene cluster of *HXK1*, *NAG1*, and *DAC1* is syntenic in all CUG clade species, with the direction of transcription of each gene conserved (37), indicating that the acquisition of these genes, while predating the divergence of this clade, is a relatively recent event. The more distantly related *Candida glabrata*, a non-CUG species, cannot utilize GlcNAc and does not alter pH when grown on this medium. Not surprisingly, the *C. glabrata* genome does not encode homologs of the GlcNAc transporter or catabolic enzymes.

10.1128/mSphere.00357-17.1 FIG S1  Other members of the CUG clade of *Candida* spp. neutralize the environmental pH. (A) CUG clade species outperform distantly related *C. glabrata* in media with both amino acids and GlcNAc. Cells were grown in YNBA with 1% Casamino Acids or 20 mM GlcNAc as the sole carbon source and assessed for pH at the specified times. (B) Growth in YNBA plus 20 mM GlcNAc only induces hyphal morphogenesis and flocculation in *C. albicans* after 8 h of incubation. Cells were washed, fixed with 2.7% paraformaldehyde, stored in 1× PBS, and then viewed using DIC microscopy at ×40 magnification. Download FIG S1, PDF file, 1.8 MB. Copyright © 2017 Vesely et al. 2017 Vesely et al. This content is distributed under the terms of the Creative Commons Attribution 4.0 International license.

### pH neutralization with GlcNAc is genetically distinct from pH neutralization with amino acids.

**FIG 2:**
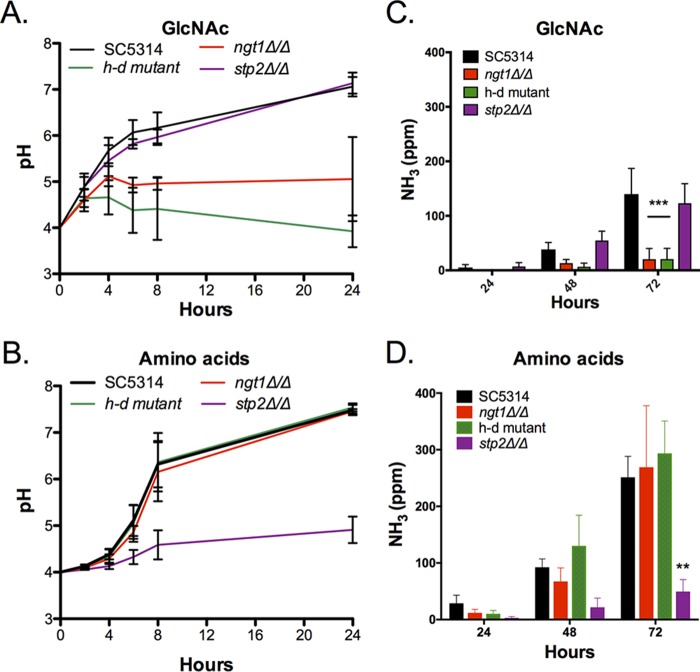
*N*-Acetylglucosamine-induced pH neutralization is dependent on its transport and catabolism but is independent of the amino-acid-based mechanism. (A and B) Wild-type and mutant strains of *C. albicans* were assessed for pH neutralization in YNBAG plus 20 mM *N*-acetylglucosamine or 1% Casamino Acids over 24 h. (C and D) Ammonia released by *C. albicans* cells during pH neutralization on solid YNBAG plus 20 mM *N*-acetylglucosamine or 1% Casamino Acids. Ammonia was detected after 24, 48, and 72 h via Nessler’s reagent. **, *P* < 0.05. Data are expressed as mean values ± SD from triplicate experiments. YNBG medium consists of minimal YNB supplemented with 0.5% glycerol.

We next sought to compare the two mechanisms of pH neutralization using a genetic approach. First, we examined a mutant strain (*hxk1Δ nag1Δ dac1Δ*) that lacks the three enzymes responsible for the conversion of GlcNAc to fructose-6-phosphate, an intermediate of glycolysis, referred to as the “*h-d* mutant” (29). When the *h-d* mutant was grown with GlcNAc as the sole carbon source, there were no appreciable changes in pH (Fig. 2A). A second mutant, lacking the GlcNAc-specific transporter Ngt1, also failed to neutralize the media, although there was a slight increase in pH (Fig. 2A), perhaps resulting from low-affinity transport through another membrane permease. Both strains exhibited robust extracellular pH neutralization when CAA was present as the carbon source, indicating that the genes responsible for transport and catabolism of GlcNAc do not affect growth or neutralization with amino acids.

To determine whether the genes required for amino-acid-driven pH neutralization affected GlcNAc-induced pH changes, we used a deletion of *STP2*, which encodes a transcription factor that regulates many genes necessary for amino acid catabolism and pH neutralization (24, 28, 38). This strain was unaltered in its ability to change the pH with GlcNAc as the carbon source, indicating that Stp2 is not required for this process (Fig. 2B). When assessed for ammonia release, the *ngt1Δ* and *h-d* mutants were impaired when grown on GlcNAc, but not amino acids, as the carbon source (Fig. 2C and D). The *stp2Δ* mutant, in contrast, showed the opposite pattern. Both results were consistent with the effects on the ambient pH of the media, although it was somewhat unexpected that GlcNAc elicited about half the concentration of ammonia relative to CAA given the comparable change in pH (Fig. 2C and D).

**FIG 3:**
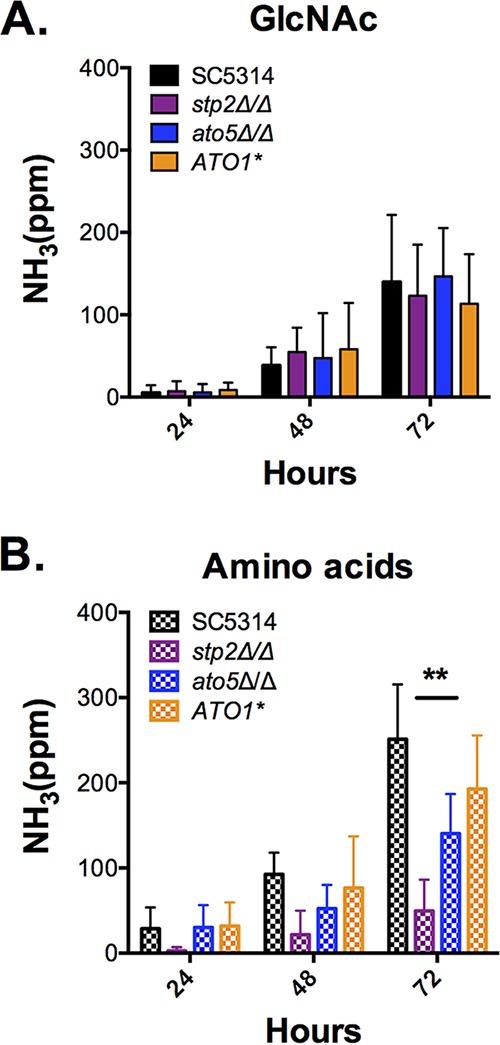
Putative ammonia transporters are not required for ammonia release during *N*-acetylglucosamine pH neutralization. Panels A and B show ammonia released by *C. albicans* cells during pH neutralization on solid YNBAG plus 20 mM *N*-acetylglucosamine (A) or 1% Casamino Acids (B). Ammonia was detected after 24, 48, and 72 h via Nessler’s reagent. **, *P* < 0.05. Data are expressed as mean values ± SD from triplicate experiments.

We have previously implicated several members of a multigene family termed *ATO* (for ammonia transport outward) in ammonia release during pH neutralization, in particular *ATO1* and *ATO5* (39). To ask whether these facilitated pH neutralization in the presence of GlcNAc, we assayed ammonia release with both amino acids and GlcNAc as the carbon source in both *ato5Δ* and ATO1* mutants. (ATO1* is a dominant-negative mutation in Ato1.) We confirmed our earlier observations in the presence of CAA (Fig. 3A); in contrast, these strains phenocopied the parental strain when GlcNAc was used as the carbon source, indicating that the *ATO* genes are not required for this process (Fig. 3B).

### Both transport and catabolism of GlcNAc are necessary for robust virulence during interactions with murine macrophages.

**FIG 4:**
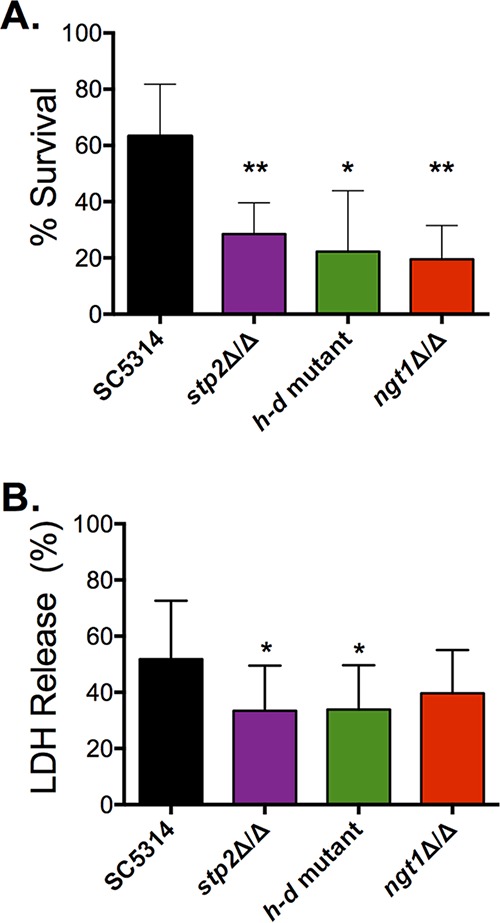
Transport and catabolism of *N*-acetylglucosamine is necessary for robust virulence during interactions with murine macrophages. (A) The survival of *stp2*Δ/Δ, *h-d*, and *ngt1*Δ/Δ strains was assessed using a modified CFU assay after incubation with RAW264.7 macrophages for 16 h. Percentage of survival is relative to the number of colonies formed for each strain in RPMI supplemented with 5% FBS–1% penicillin/streptomycin alone. (B) Macrophage cytotoxicity was measured via the detection of released lactate dehydrogenase (LDH) from RAW264.7 murine macrophages after 16 h of coincubation with each indicated strain. Percentage of release is relative to chemically lysed macrophage controls. *, *P* < 0.05; **, *P* < 0.01. Data are expressed as mean values ± SD from triplicate experiments.

The *h-d* mutant has a reduced virulence phenotype in the murine model of disseminated candidiasis (29, 40). We sought to investigate if this may be due to defects in interactions with murine innate immune cells. For this reason, we examined *C. albicans* survival upon phagocytosis by RAW264.7 cells as well as the ability of *C. albicans* to elicit cell damage. When assessed for survival after phagocytosis, it was clear that the *ngt1Δ* and *h-d* mutants had diminished survival compared to the parental strain (Fig. 4A). When the supernatant was examined for levels of lactate dehydrogenase (LDH), a proxy for macrophage membrane damage, it was also clear that the *ngt1Δ* and *h-d* mutants induced less cytotoxicity than the parent strain (Fig. 4B). The magnitudes of the defects in survival and macrophage damage were similar to those observed in the *stp2Δ* strain, suggesting that both GlcNAc and amino acids are relevant nutrients in the macrophage phagosome. This is consistent with the modest impairment of virulence observed with both the *stp2*Δ and *h-d* mutants in the mouse disseminated candidiasis model (24, 29).

**FIG 5:**
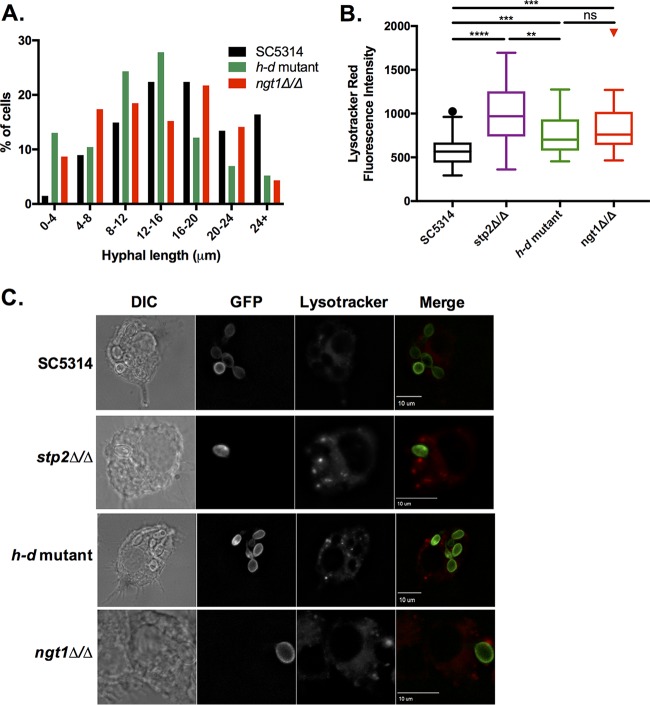
Mutants impaired in *N-*acetylglucosamine transport and catabolism display morphological and pH neutralization defects within macrophages. (A) GFP-tagged *C. albicans* strains were cocultured with RAW264.7 macrophages in RPMI medium for 2 h. Samples were counterstained with calcofluor white to identify internalized cells, and hyphal length was measured using Slidebook 6 software. (B) RAW264.7 macrophages preloaded with the acidophilic dye LysoTracker red were cocultured with SC5314, the *stp2Δ* mutant, the *h-d* mutant, or the *ngt1Δ* mutant expressing a C-terminal Pma1-GFP fusion. Phagosomal pH was measured by quantifying LysoTracker red fluorescent intensity using Slidebook 6 software. **, *P* < 0.01; ***, *P* < 0.001; ****, *P* < 0.0001; ns, not significant. The boxes represent cells with fluorescence intensities from 25% to 75% and the whiskers are 5% to 95%. At least 50 cells were counted per strain. (C) Representative images of each strain. *C. albicans* fluorescence results with GFP, Lysotracker red, and mCherry are shown.

The reduced fitness of the *stp2Δ* mutant strain during interactions with macrophages is associated with impaired germination of phagocytosed cells (24). To ask whether this was also the case for the GlcNAc catabolic machinery, we assayed the morphology of *ngt1Δ* and *h-d* mutant cells upon phagocytosis by murine macrophages. We measured the length of the germ tube (if any) from the body of the mother yeast cell to the tip of the projection. The *ngt1Δ* and *h-d* mutant strains had a higher proportion of cells with either shorter hyphal projections or that had not germinated relative to the wild-type control (Fig. 5A). These results indicate that utilization of GlcNAc contributes to hyphal growth of phagocytosed cells, although the effects on morphology are less pronounced than when amino acid catabolism is impaired via mutation of *STP2*.

### *ngt1Δ* and *h-d* strains occupy an acidic phagolysosome.

We previously demonstrated that phagocytosed *C. albicans* cells inhabit a compartment (phagosome or phagolysosome) of neutral rather than the expected acidic pH and that the SPS amino acid sensor, Stp2, Ato1, and Ato5 are required to maintain neutrality (21, 24, 39). Furthermore, neutral pH induces hyphal germination (24). The macrophage-associated phenotypes observed with the GlcNAc mutants are consistent with occupancy in an acidic phagolysosome. To test this, we utilized the acidotropic dye LysoTracker red (LR), which accumulates and fluoresces in acidic organelles. As previously published for the *stp2Δ* mutant, the *ngt1Δ* and *h-d* mutant cells colocalize with LR to a greater extent than the parental strain, indicating these cells are present in an acidic compartment (Fig. 5B and C). We measured LR fluorescence intensity immediately outside the fungal cells, in the lumen of the phagosome, as described previously (21, 39), and found that while the *ngt1Δ* and *h-d* mutant cells were not as impaired as the *stp2*Δ cells, there was a statistically significant increase in LR staining relative to phagosomes containing wild-type cells, indicating that these mutants are in a more acidic environment.

## DISCUSSION

This study elaborates upon the finding that *C. albicans* can utilize *N*-acetylglucosamine (GlcNAc) to manipulate the environmental pH (29). This phenomenon bears similarities to the extracellular neutralization that results from catabolism of amino acids (28), in the overall magnitude of the pH change, and the excretion of ammonia as a driving force. By genetically blocking the utilization of GlcNAc, either by disrupting the transport from the extracellular environment (*ngt1Δ*) or by abolishing the enzymes responsible for its catabolism (*hxk1Δ*,* nag1Δ*,* dac1Δ*, or *h-d*), we have shown that utilization of GlcNAc is required for robust neutralization, as opposed to a strictly signaling role as described for GlcNAc-induced morphogenesis (41). In contrast, none of the described mutations that abolish amino-acid-induced pH changes, including in mutants lacking the Stp2 transcription factor or the Ato1 or Ato5 putative ammonia/acetate transporters (21, 24, 39), affect neutralization in the presence of GlcNAc. Like these mutants, however, the *ngt1Δ* and *h-d* mutants are impaired in several measures of fitness within macrophages as a result of occupying a more acidic phagolysosome. Thus, despite some similarities, the amino-acid-induced neutralization and GlcNAc-induced neutralization occur by distinct processes.

In addition to amino acids, *C. albicans* can raise the environmental pH when utilizing carboxylic acids, such as α-ketoglutarate, pyruvate, or lactate, as a carbon source (15). This pH change is not accompanied by hyphal morphogenesis or detectable ammonia release and thus differs from either the amino-acid- or GlcNAc-induced mechanism. Like these processes, however, cells impaired in carboxylic acid utilization are less able to resist killing by macrophages (15). Thus, *C. albicans* has at least three independent pathways through which it can manipulate the pH of its surroundings, each based on nonpreferred nutrients that appear to be available in the macrophage phagosome. There is evidence that combining amino acid and carboxylic acid mutations results in additive defects in macrophage interactions (15). We speculate that disabling all three systems would reduce fungal survival after phagocytosis to a much greater degree than was observed in any of the single mutants.

The comparison of growth and pH in the presence of amino acids versus GlcNAc highlights two questions regarding the mechanisms of pH change. First, the rise in pH is more rapid with GlcNAc than with amino acids, despite producing less excreted ammonia. This might reflect a shorter lag time in the transition from glucose-grown precursors to GlcNAc, which is metabolized largely through glycolysis, than for amino acids, which entails a switch to gluconeogenesis. Alternatively, the buffering capacity of media with GlcNAc might be less than for amino acids, making the released ammonia more effective. Second, hyphal growth in phagocytosed cells of the *ngt1Δ* and *h-d* mutants is only modestly reduced relative to the severe defect of a strain lacking *STP2* (24). GlcNAc is a potent morphogenetic inducer, sensed via an intracellular mechanism that does not require its catabolism through Hxk1 (41): thus, the inability to break down this compound might actually increase its concentration relative to phagosomes containing wild-type strains, potentiating signaling.

Evidence is mounting that *C. albicans* uses nonglucose carbon sources as a signal of the host environment. The Brown lab has convincingly demonstrated that growth of cells on lactate, an abundant carboxylic acid found in mammalian niches, affects cell wall structure, adhesion, and drug resistance of *C. albicans*, and these combine to alter recognition by macrophages (25–27). GlcNAc is also abundant throughout the mammalian host and triggers a hyphal morphogenetic program in *C. albicans* and in the dimorphic fungi *Histoplasma capsulatum* and *Blastomyces dermatitidis*; catabolism is not required for this effect in any of these species (41, 42). In *C. albicans*, the cyclic AMP (cAMP)-dependent protein kinase A pathway is required for GlcNAc-induced morphogenesis, but not for its catabolism, while a newly identified transcription factor, Ron1, regulates catabolism and morphogenesis (43, 44). GlcNAc has emerged as a signal of the host environment in pathogenic bacteria as well, including *Pseudomonas aeruginosa*, where it regulates production of phenazines, and *Escherichia coli*, where it promotes adhesion and biofilm formation via expression of fimbriae and curli fibers (35, 45, 46). Interestingly, host cells also use GlcNAc as a signaling molecule. A recent study reported that GlcNAc released from breakdown of bacterial cell wall peptidoglycan in the phagosome can trigger NLRP3 inflammasome activation in macrophages (47). Thus, a variety of species have evolved mechanisms to sense GlcNAc to regulate virulence and colonization determinants.

The macrophage-*Candida* coculture system has been a valuable model for identifying factors that mediate systemic virulence. Indeed, both the *stp2Δ* and GlcNAc-deficient mutants are attenuated in the mouse disseminated hematogenous model (24, 29, 40). Several strains with impaired ability to utilize carboxylic acids are also attenuated in animals or in cell culture models (9, 15, 48, 49). This reinforces the idea that many niches in the mammalian host are poor in glucose but replete in other nutrients. Some of these niches, including parts of the gastrointestinal tract, oral cavity, and vagina, have regions of low ambient pH. The role of pH modulation via alternative carbon metabolism by *C. albicans* in colonization and/or infection at these sites remains to be seen.

## MATERIALS AND METHODS

### Culture procedures.

*C. albicans* strains were grown in YPD medium (1% yeast extract, 2% peptone, 2% glucose, with or without 2% agar for solid or liquid medium) routinely before all experiments. Experiments that required a change in carbon source utilized a minimal yeast nitrogen base (YNB) medium (YNBAG) with the specified carbon source and 0.5% allantoin as the nitrogen source plus 0.5% glycerol. The use of glycerol in this medium is to allow for some growth support of genetic mutant strains; glycerol does not affect pH neutralization (21, 28). The media were adjusted to a starting pH of 4 using HCl. The murine macrophage cell line RAW264.7 was propagated routinely in RPMI 1640 with glutamate, 10% fetal bovine serum, and 1% penicillin–streptomycin in a 5% CO_2_ incubator. Macrophages were used in experiments between passages 7 and 16. Cell counting was performed by a Countess II cell counter (Thermo Fisher) when required. The *C. albicans* strains that were used in this study are described in Table 1. Derivatives of the *ngt1Δ*, *h-d*, and *stp2Δ* mutants with a plasma membrane-localized green fluorescent protein (GFP) to facilitate the LysoTracker quantification experiments were generated by integrating a *PMA1-GFP* translational fusion, as described previously (21).

**TABLE 1:** *C. albicans* strains used in this study

Strain name	Relevant genotype	Complete genotype	Reference
SC5314	Wild type	Prototroph	50
SVC17	*stp2*Δ	*stp2*Δ::*FRT*/*stp2*Δ::*FRT*	24
HDC27	*ato5*Δ	*ato5*Δ::*FRT*/*ato5*Δ::*FRT*	39
Can572	*ATO1*^*G53D*^ (“ATO1*”)	*ura3*/*ura3 RPS10*/*rps10*::*Clp10-ACT1p-ATO1*^*G53D*^	28
YJA3	*ngt1Δ*	*ngt1*Δ::*ARG4*/*ngt1*Δ::*HIS1 ura3*Δ::λ*imm*^434^/*URA3 his1*::*hisG*/*his1*::*hisG arg4*::*hisG*/*arg4*::*hisG*	51
AG738	*hxkΔ nag1Δ dac1Δ* (“*h-d* mutant”)	(*hxk1 nag1 dac1*)::*ARG4*/(*hxk1 nag1*Δ *dac1*Δ)::*URA3 HIS1*/*his1*::*hisG ura3*Δ::λ*imm*^434^/*ura3*Δ:: λ*imm*^434^* arg4*::*hisG*/*arg4*::*hisG*	28
CaPM57	SC5314 *Pma1-GFP*	*PMA1*/*PMA1*::*PMA1-GFP*	21
CaPM61	*stp2*Δ/Δ *Pma1-GFP*	*stp2*Δ::*FRT*/*stp2*Δ::*FRT PMA1*/*PMA1*::*PMA1-GFP*	21
RW01	*ngt1*Δ/Δ *Pma1-GFP*	*ngt1*Δ::*ARG4*/*ngt1*Δ::*HIS1 ura3*Δ::λ*imm*^434^/*URA3 his1*::*hisG*/*his1*::*hisG arg4*::*hisG*/*arg4*::*hisG*::*FRT* *PMA1*/*PMA1*::*PMA1-GFP*	This study
RW02	*hxkΔ nag1Δ dac1Δ Pma1-GFP*	(*hxk1 nag1 dac1*)::*ARG4*/(*hxk1 nag1*Δ *dac1*Δ)::*URA3 HIS1*/*his1*::*hisG ura3*Δ::λ*imm*^434^/*ura3*Δ:: λ*imm*^434^* arg4*::*hisG*/*arg4*::*hisG*::*FRT PMA1*/*PMA1*::*PMA1-GFP*	This study

### pH neutralization assay.

Wild-type and mutant strains of *C. albicans* were assessed for pH neutralization in YNBG plus 20 mM *N*-acetylglucosamine or 1% Casamino Acids over 24 h. All *C. albicans* cultures were grown in YPD, rolling overnight in a 30°C incubator. Cultures were washed 3 times with double-distilled water (ddH_2_O) for all pH neutralization and ammonia release experiments to wash away remaining YPD nutrients. Washed cultures were inoculated to achieve a starting optical density at 600 nm (OD_600_) of 0.2. The growth and pH of the cultures were assessed at the indicated times using a standard spectrophotometer (OD_600_) or pH electrode, as described previously (28).

### Ammonia release detection.

Ammonia release from neutralizing colonies was detected as described previously (28). Briefly, 3 µl of *C. albicans* cultures adjusted to OD_600_ of 1 was inoculated onto solid YNBAG plus 20 mM *N*-acetylglucosamine or 1% Casamino Acids. Ammonia was detected after 24, 48, and 72 h via Nessler’s reagent from a 10% citric acid trap in each petri dish. Absorbance was read at 405 nm in an automatic plate reader (BioTek Synergy H4).

### Endpoint survival assay.

To assess survival of *C. albicans* strains in coculture with macrophages, we used a modified endpoint dilution assay (24). To do so, RAW264.7 murine macrophages were inoculated in a 96-well plate at a concentration of 2.5 × 10^4^ cells/ml and incubated overnight to reach a concentration of 5 × 10^4^ cells/ml. *C. albicans* strains were grown overnight in YPD at 30°C, collected by centrifugation, washed three times with phosphate-buffered saline (PBS), and diluted in PBS. Cocultures were inoculated starting at a multiplicity of infection (MOI) of 1:1 and serially diluted 5 times before incubation for a total of 16 h postinoculation. Graphs represent a visual count of colony-forming units (CFU) in row 5 of the serial dilution compared to *C. albicans* incubated without macrophages present.

### Macrophage cytotoxicity.

Fungus-induced damage to the macrophages was assayed by detection of lactate dehydrogenase (LDH) as described previously (24) using the Cytotox 96 kit (Promega). RAW264.7 murine macrophages were inoculated in a 96-well plate at a concentration of 2.5 × 10^4^ cells/ml and incubated overnight to reach a concentration of 5 × 10^4^ cells/ml. *C. albicans* strains were prepared as described above, inoculated at an MOI of 1:1, and incubated in RPMI at 37°C in 5% CO_2_ for 16 h. The percentage of release is relative to chemically lysed macrophage controls, accounting for spontaneous release during incubation time. Absorbance was read at 450 nm in an automatic plate reader (BioTek Synergy H4).

### LysoTracker red pH assessment and morphological surveillance.

SC5314, *h-d* mutant, *ngt1Δ*/*Δ*, and *stp2Δ*/*Δ* strains were modified to feature a GFP-tagged Pma1, as previously described (21). RAW264.7 murine macrophages were inoculated in 8-well slides at a concentration of 2.5 × 10^4^ cells/ml and incubated overnight to reach a concentration of 5 × 10^4^ cells/ml. For LysoTracker experiments, macrophages were pretreated with 0.1 mM LysoTracker red for 1 h prior to inoculation. *C. albicans* cultures were prepared as described above and inoculated at an MOI ratio of 3:1 prior to incubation for 45 min in a 5% CO_2_ incubator at 37°C. For morphological surveillance of *C. albicans* after phagocytosis, cocultures were incubated for 1, 2, and 3 h. Cells were then stained with 1:300 calcofluor white very briefly, washed three times with PBS, and fixed with 2.7% paraformaldehyde at 37°C for 15 min before storage at 4°C in PBS. Cocultures were imaged under ×60 magnification by differential inference contrast (DIC) and corresponding fluorescence spectrums. Image analysis was performed using SlideBook 6.0. The ratio of LysoTracker signal to GFP was obtained as previously described (21).

### Statistical analyses.

All statistical analyses were performed with Prism 6.
