# Supplementary material for: N-Acetylglucosamine Metabolism Promotes Survival of Candida albicans in the Phagosome
Source: mSphere. 2017 Sep 6;2(5):e00357-17. doi: 10.1128/mSphere.00357-17 (PMC5588037; doi:10.1128/mSphere.00357-17)

SUPPLEMENTAL MATERIAL

Supplemental Figure 1: **Other members of the CUG clade of *Candida spp*. neutralize the environmental pH.** A) CUG clade species outperform distantly related *C. glabrata* in media with both amino acids and GlcNAc. Cells were grown in YNBA with 1% Casamino acids or 20 mM GlcNAc as the sole carbon source and assessed for pH at the specified times. B) Growth in YNBA + 20 mM GlcNAc only induces hyphal morphogenesis and flocculation in *C. albicans* after 8 hours of incubation. Cells were washed, fixed with 2.7% paraformaldehyde, stored in 1xPBS, then viewed using DIC microscopy at 40x magnification.

A.

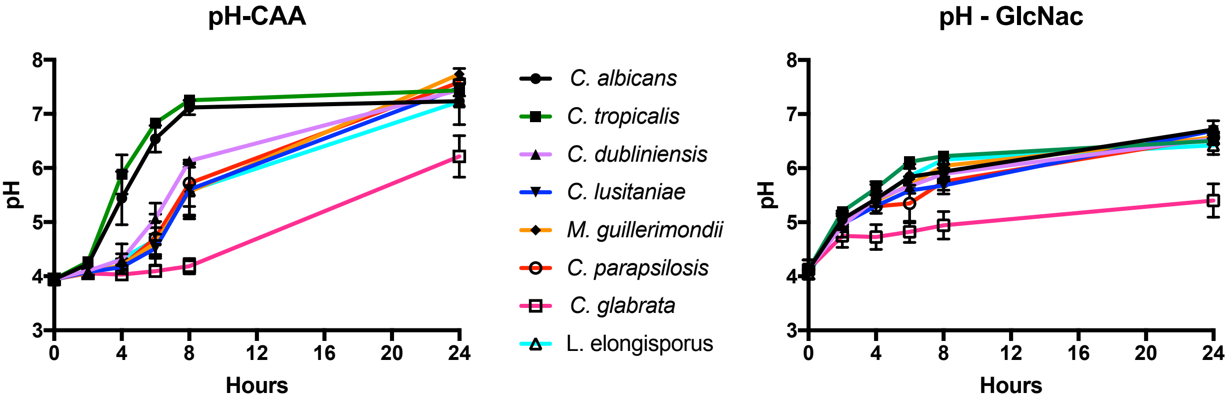

B.

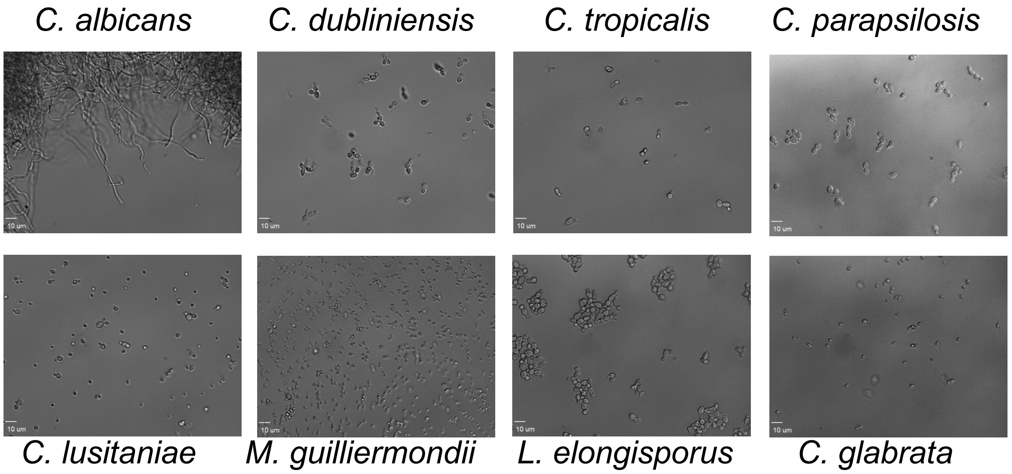

Supplement: FIG S1 [file sph005172356sf1.pdf]
